# Uncertainty-Aware Knowledge Distillation for Collision Identification of Collaborative Robots

**DOI:** 10.3390/s21196674

**Published:** 2021-10-08

**Authors:** Wookyong Kwon, Yongsik Jin, Sang Jun Lee

**Affiliations:** 1 Daegu-Gyeongbuk Research Center, Electronics and Telecommunications Research Institute (ETRI), Daegu 42994, Korea; wkwon@etri.re.kr (W.K.); yongsik@etri.re.kr (Y.J.); 2 Division of Electronic Engineering, Jeonbuk National University, 567 Baekje-daero, Deokjin-gu, Jeonju 54896, Korea

**Keywords:** collision identification, collaborative robot, deep learning, uncertainty estimation, knowledge distillation

## Abstract

Human-robot interaction has received a lot of attention as collaborative robots became widely utilized in many industrial fields. Among techniques for human-robot interaction, collision identification is an indispensable element in collaborative robots to prevent fatal accidents. This paper proposes a deep learning method for identifying external collisions in 6-DoF articulated robots. The proposed method expands the idea of CollisionNet, which was previously proposed for collision detection, to identify the locations of external forces. The key contribution of this paper is uncertainty-aware knowledge distillation for improving the accuracy of a deep neural network. Sample-level uncertainties are estimated from a teacher network, and larger penalties are imposed for uncertain samples during the training of a student network. Experiments demonstrate that the proposed method is effective for improving the performance of collision identification.

## 1. Introduction

With the increasing demands of collaborative tasks between humans and robots, the research on human–robot interaction has received great attention from researchers and engineers in the field of robotics [1]. Robots that can collaborate with humans are called collaborative robots (cobots), and cobots differ from conventional industrial robots in that they do not require a fence to prevent access. Previously, the application of robots is limited to performing simple and repetitive tasks in well-structured and standardized environments such as factories and warehouses. However, the development of sensing and control technologies has significantly expanded the area of application of cobots [2], and they are beginning to be applied to several tasks around us. More specifically, their applications have been diversified from traditional automated manufacturing and logistics industries to more general tasks such as medical [3], service [4,5], food and beverage industries [6], and these tasks require more elaborate sensing and complicated control techniques. Furthermore, with the development of intelligent algorithms including intention estimation [7] and gesture recognition [8], cobots can be utilized in wider application areas.

In general, robots have advantages over humans in repetitive tasks, and humans are better at making comprehensive decisions and judgments. Therefore, human–robot collaboration possibly increases the efficiency of intelligent systems through complementary synergies. As the scope of robotics applications gradually expands through collaborative work, interaction with humans or unstructured environments has become an important technical issue, which requires the implementation of advanced perception and control algorithms. Especially, collision detection and identification techniques are indispensable elements to improve the safety and reliability of collaborative robots [9,10].

To perform cooperative tasks with the aid of human–robot interactions, several studies have been carried out to detect and identify robot collisions for the safety of workers [11]. Previous work can be categorized into two approaches: the first category is the study on the control of collaborative robots by predicting possible collisions and the other is the study of responses after impacts. While collision avoidance is more advantageous in terms of safety [12], this approach inevitably requires additional camera sensors for action recognition of coworkers or 3D reconstruction of surrounding environments [13]. Furthermore, it is difficult to completely avoid abrupt and unpredictable collisions. Therefore, techniques for collision identification are essential to improve the safety and reliability of collaborative robots.

Collision detection algorithms investigate external forces [14] or currents [15] to determine whether a true collision has occurred on an articulated robot. A key element in the procedure of collision detection is the estimatation of external torques. A major approach to estimating external torques is utilizing torque sensor signals to compute internal joint torques based on the physical dynamics of robots, and several other methods to construct momentum observers to estimate external torques without the use of torque sensors. The method that does not use torque sensors is called sensorless external force estimation, and an elaborate modeling of the observer and filter is essential for the precise estimation of external forces [16,17,18,19]. External forces are further processed by a thresholding method [20] or classification algorithm [21], to determine whether a collision has occurred. Recently, deep-learning-based methods have outperformed traditional model-based methods in detecting collisions [22]. Beyond collision detection, the identification of collision locations is beneficial for the construction of more reliable collaborate robots, by making them react appropriately in collision situations.

To ensure the proper responses of collaborative robots in cases of collisions, it is necessary to identify collision locations. The collision identification technique can be defined as a multiclass classification of time series sensor data according to collision locations. In early studies, collision identification was mainly based on the elaborate modeling of filters [23] and observers [24], and a frequency domain analysis was conducted to improve the accuracy of collision identification [25]. To address the classification problem, machine learning techniques, which were employed to analyze time series data, have also been applied to collision identification [26]. Recently, support vector machines [27] and probabilistic methods [28] were applied to improve the reliability of collision identification systems. In [29], the collision identification performance was improved by utilizing additional, sensors such as inertial measurement units, and analyzing their vibration features.

In this paper, we propose a method that can identify collisions on articulated robots by utilizing deep neural networks for joint sensor signals. Collision identification refers to a technique that not only detects the occurrence of a collision, but also determines its location. Recently, a collision detection method was proposed by Heo et al. [22]; we extend this existing method for collision identification and improve the robustness of the deep neural network. To improve the performance of the collision identification system, we construct a deeper network, which is called a teacher network, to distill its probabilistic knowledge to a student network. In the process of distilling knowledge, we employ the uncertainties of the teacher network to focus on learning difficult examples, mostly collision samples. This paper is organized as follows. Section 2 presents related work, Section 3 explains collision modeling and data collection, and Section 4 presents the proposed method. Section 5 and Section 6 presents the experimental results and conclusion, respectively.

## 2. Related Work

### 2.1. Deep Learning Methods for Collision Identification of Collaborative Robots

Collision detection is a key technology to ensure the safety and reliability of collaborative robots. Although most previous methods were based on the mathematical modeling of robots [30,31,32], recently, deep learning methods have shown promising results for this goal. Min et al. [33] estimated vibration features based on the physical modeling of robots and utilized neural networks for collision identification. Xu et al. [34] combined neural networks and nonlinear disturbance observer for collision detection. Park et al. [35] combined a convolutional neural network and support vector machine to detect collisions, and Heo et al. [22] employed causal convolutions, which were previously utilized for auto-regressive models in WaveNet [36] to detect collisions based on joint sensor signals including torque, position, and velocity. Maceira et al. [37] employed recurrent neural networks to infer the intentions of external forces in collaborative tasks, and Czubenko et al. [38] proposed an MC-LSTM, which combines convolutions and recurrent layers for collision detection. Mohammadi et al. [13] utilized external vision sensors to further recognize human actions and collisions.

### 2.2. Knowledge Distillation

Knowledge distillation was proposed by Hinton et al. [39] to train a student network with the aid of a deeper network, which is called a teacher network. Probabilistic responses of the teacher network are beneficial to improve the accuracy of the student network because the probabilities of false categories were also utilized during knowledge distillation. Although most early methods directly distill the logits of a teacher network, Park et al. [40] utilized the logits’ relations, and Meng et al. [41] proposed a conditional teacher–student learning framework. Furthermore, knowledge from intermediate feature maps was distilled for network minimization [42] and performance improvement [43,44]. Knowledge distillation has been employed in various applications such as object detection [45], semantic segmentation [46], domain adaptation [47], and defense for adversarial examples [48]. Recently, the teacher–student learning framework has been applied with other advanced learning methodologies such as adversarial learning [49] and semi-supervised learning [50].

### 2.3. Uncertainty Estimation

Uncertainty plays an important role in interpreting the reliability of machine learning models and their predictions. Probabilistic approaches and Bayesian methods have been regarded as useful mathematical tools to quantify predictive uncertainties [51]. Recently, Gal and Ghahramani proposed Monte Carlo dropout (MC-dropout) [52], which can be interpreted as an approximate Bayesian inference of deep Gaussian processes, by utilizing dropout [53] at test time. Lakshminarayanan et al. [54] proposed deep ensembles for the better quantification of uncertainties, and Amersfoort et al. [55] proposed deterministic uncertainty quantification, which is based on a single model to address the problem of computational cost of MC-dropout and deep ensembles. Uncertainties have been utilized to quantify network confidences [56], selecting out-of-distribution samples [57], and improving the performance of deep neural networks [58,59], in various application areas such as medical image analysis [60] and autonomous driving [61].

## 3. Collision Modeling and Data Collection

### 3.1. Mathematical Modelling of Collisions

This section explains the mathematical modeling of dynamic equations for 6 Degrees of Freedom (DoF) articulated robots. In order to operate a robot through a desired trajectory and move it to a target position, precise control torque is required for each joint motor, and the control torque can be represented as the following dynamic equation:

(1) $$\tau =M\left(q\right)\ddot{q}+C\left(q,\dot{q}\right)\dot{q}+g\left(q\right),$$

where $\tau \in R^{n}$ is the control torque, $M\left(q\right)\in R^{n\times n}$ is the inertia matrix of the articulated robot, $C\left(q,\dot{q}\right)\in R^{n\times n}$ is the matrix of Coriolis and Centrifugal torques, $g\left(q\right)\in R^{n}$ is the vector of gravitational torques, and *q*, $\dot{q}$, $\ddot{q}$ are the angular position, velocity, and acceleration of each joint, respectively. The dynamic equation can be obtained through the Newton–Euler method or the Euler–Lagrange equation using the mechanical and physical information of the robot. Since the dynamic equation of the robot is given as (1), in the absence of external force, external torques can be computed by subtracting the control torques from measured torques.

When a joint torque sensor is installed onto each joint, the torque generated on each joint due to external force is given as follows:

(2) $$\tau_{ext}=\tau_{s}-\tau ,$$

where $\tau_{ext}$ is the external torques generated onto each joint due to a collision, and $\tau_{s}$ is torque values measured by joint torque sensors. The external torque can be precisely estimated under an accurate estimation of robot dynamics and physical parameters of the articulated robot such as the mass and center of mass of each link.

In robots that are not equipped with a joint torque sensor, sensorless methods are utilized to estimate external torques. Sensorless methods are basically based on the current signal of each joint motor, and an additional state variable $p=M\left(q\right)\dot{q}$ is defined to reformulate the dynamic equation as follows:

(3) $$\dot{p}=C{\left(q,\dot{q}\right)}^{⊤}\dot{q}-g\left(q\right)-f\left(q,\dot{q}\right)+\tau_{m},$$

where $f(q,\dot{q})$ is the friction matrix, and $\tau_{m}$ is the motor torque. In the case of the sensorless method, it is necessary to obtain the transmitted torque from the motor to the link to estimate the collision torque. Therefore, the friction must additionally be considered in the existing robot dynamics equation. A main issue in sensorless external torque estimation is the elaborate design of observer and filter under the dynamic Equation (3), and the effect of disturbance can be reduced using momentum state variables. Due to the effect of noise and nonlinear frictional force, sensorless methods are generally less precise in the estimation of external torques compared to methods that utilize joint torque sensors. Through the methods mentioned above, it is possible to obtain the torques generated in each joint due to the collision of the robot. Then, the collision identification algorithm can determine collision locations from joint torques obtained through sensor or sensorless methods.

### 3.2. Data Collection and Labeling

**Figure 1:**
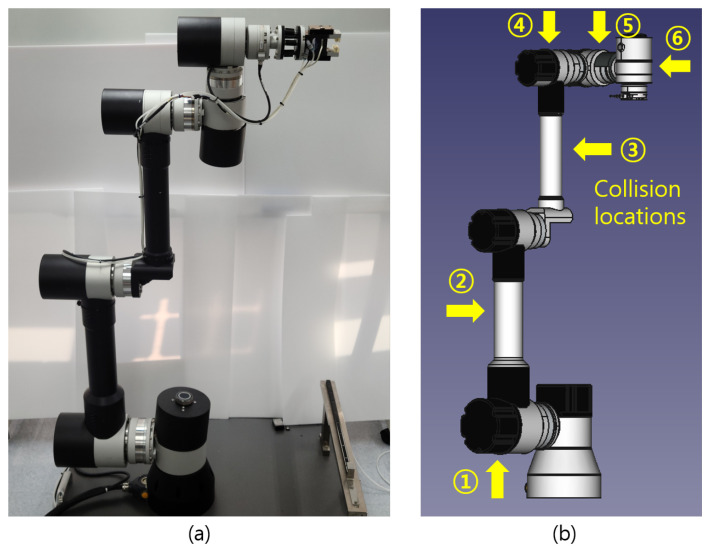
The definition of labels. (**a**) presents 6-DoF articulated robot, and (**b**) presents the definition of categories; yellow arrows in (**b**) indicate categorical labels according to collision locations.

Figure 1a presents the 6-DoF articulated robot to collect sensor data, which include the information of joint torque, current, angular position, and angular velocity. The Denavit–Hartenberg parameters of the articulated robot are presented in [62]. From the 6-DoF articulated robot, joint sensor signals were obtained with the sampling rate of 1 kHz, and a data sample collected at time *t* can be expressed as

(4) $$x_{t}={\left[\tau_{t}^{⊤},i_{t}^{⊤},\theta_{t}^{⊤},w_{t}^{⊤}\right]}^{⊤}\in R^{24},$$

where $\tau_{t}$, $i_{t}$, $\theta_{t}$, $w_{t}$ are six-dimensional vectors corresponding to torque, current, angular position, and angular velocity, respectively; the *i*-th components of these vectors indicate the sensor signals obtained at the *i*-th joint. Figure 1b shows the definition of collision categories according to collision locations. Collisions were generated at six locations, and in the case of no collision, which refers to the normal state, a label of 0 was assigned. In the case of a collision, a categorical label corresponding to the location was assigned to generate ground truth data.

**Figure 2:**
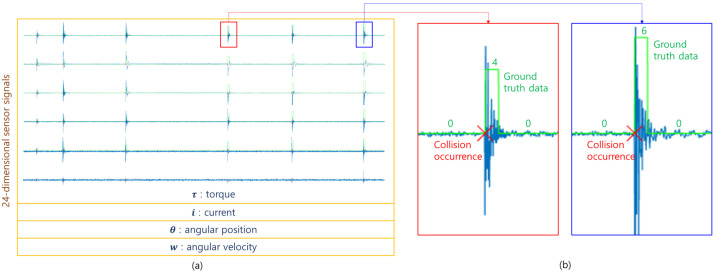
Examples of sensor signals and ground truth data. (**a**) shows a part of the acquired sensor signals, and (**b**) presents examples of generated ground truth data around collision occurrences. Green lines with numbers in (**b**) indicate labeled categories in the ground truth data.

Joint sensor data were collected, along with collision time and category, by applying intentional collisions at different locations. The collision time and category were converted into ground truth data which have an identical length to the corresponding sensor signals, as shown in Figure 2. For a collision occurrence, the corresponding category was assigned to 0.2 s of data samples from the collision time; each collision is represented as 200 collision samples in the ground truth data. We collected joint sensor signals for 5586 intentional collisions along with their ground truth data; the number of collisions, which were applied to different locations, is equal. This dataset was divided into training, validation, and test sets with the ratio of 70%, 10%, and 20%, as presented in Table 1.

## 4. Proposed Method

This section presents the proposed method for the collision identification of articulated robots. Firstly, two neural network architectures are presented; one of them is a student network and the other architecture is a teacher network for knowledge distillation. The second part explains the proposed knowledge distillation method, which considers the predictive uncertainties of the teacher network. Lastly, a post-processing is utilized to improve the robustness of the proposed algorithm by reducing noise in network predictions.

### 4.1. Network Architectures

This paper employs the network architecture presented by Heo et al. [22] as a base network model. Heo et al. [22] proposed a deep neural network, called CollisionNet, to detect collisions in articulated robots. Its architecture is composed of causal convolutions to reduce detection delay and dilated convolutions to achieve large receptive fields. We modeled the base network by modifying the last fully connected layer in CollisionNet to conduct multiclass classification and identify collision locations. The base network is composed of seven convolution layers and three fully connected layers, and its details are identical to CollisionNet except the last layer; convolution filters with the size of 3 are utilized for both regular and dilated convolutions, the depth of the intermediate features is increased from 128 to 512, and the dilation ratio is increased by a factor of two. The architecture of the base network is identically utilized as a student network in the process of knowledge distillation.

**Figure 3:**
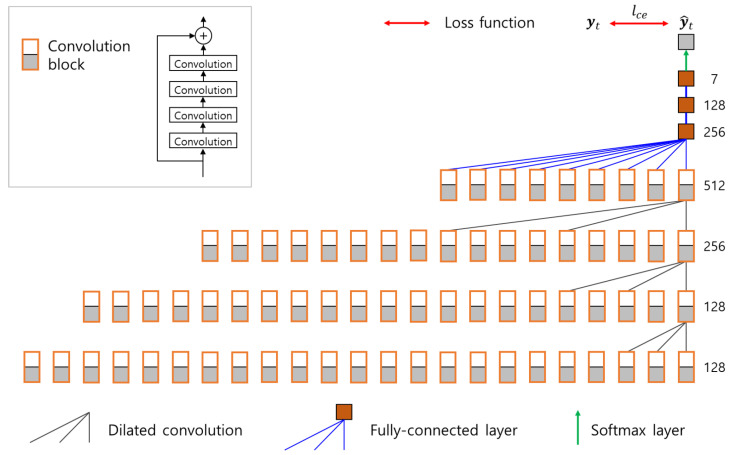
The architecture of the teacher network.

Figure 3 shows the architecture of the teacher network. To construct the teacher network, three regular convolutions in the base network are replaced into convolution blocks. A convolution block contains four convolution layers with a skip connection, and therefore, the number of parametric layers in the teacher network increases to 19. The number of channels in the second and third convolution layers in a convolution block are identical to the number of output channels of the corresponding regular convolution layers. The number of trainable parameters in the teacher network is 6.63 million; therefore, it has more capacity to learn the training data compared to the base network, which has 2.79 million parameters. Dropout layers with a dropout ratio of 0.5 are added to the fully connected layers in the teacher network, and Monte Carlo samples from the teacher network are acquired by applying dropout at the test time.

### 4.2. Uncertainty-Aware Knowledge Distillation

The teacher network is trained with the cross-entropy loss between the softmax prediction ${\hat{y}}_{T}$ and its one hot encoded label y. The *i*-th component of ${\hat{y}}_{T}$ indicates the predicted probability that the input sample belongs to the *i*-th category. In our case, seven categories exist, which contain the normal state and six possible collision locations. The loss function for the training of the teacher network is defined as

(5) $$l_{ce}\left(y,{\hat{y}}_{T}\right)=-\sum_{i}y_{i}\log\left({\hat{y}}_{T,i}\right),$$

where $y_{i}$ and ${\hat{y}}_{T,i}$ are the *i*-th components of y and ${\hat{y}}_{T}$, respectively.

After training the teacher network, *K* logits, ${\hat{z}}_{T}^{1}$, ⋯, ${\hat{z}}_{T}^{K}$ are obtained from an input sample by utilizing MC-dropout [52]. These logits are computed by randomly ignoring 50% of neurons in the fully connected layers in the teacher network. Based on the *K* logits of the teacher network, the *i*-th component of the uncertainty vector is computed by

(6) $$u_{i}=\frac{1}{K}\sum_{k}{\left({\hat{z}}_{T,i}^{k}-{\bar{z}}_{T,i}\right)}^{2},$$

where ${\bar{z}}_{T,i}$ is the *i*-th component of the averaged logit ${\bar{z}}_{T}$, which is computed by

(7) $${\bar{z}}_{T}=\frac{1}{K}\sum_{k}{\hat{z}}_{T}^{k}.$$

The uncertainty $u_{i}$ is the variance of logits; therefore, the value of the uncertainty increases as distances between the logits increase.

The total loss L for the training of the student network is composed of two loss functions, as follows:

(8) $$L=l_{ce}\left(y,{\hat{y}}_{S}\right)+l_{kd}\left({\bar{z}}_{T},{\hat{z}}_{S},u\right),$$

where $l_{ce}\left(y,{\hat{y}}_{S}\right)$ is the cross-entropy loss between the softmax prediction of the student network and its corresponding label, u is the uncertainty vector whose *i*-th component is $u_{i}$, and $l_{kd}\left({\bar{z}}_{T},{\hat{z}}_{S},u\right)$ is the uncertainty-aware knowledge distillation loss. The knowledge distillation loss os obtained by computing uncertainty-weighted Kullback–Leibler divergence (KL divergence) between $\sigma ({\hat{z}}_{S},T)$ and $\sigma ({\bar{z}}_{T},T)$, as follows:

(9) $$l_{kd}\left({\bar{z}}_{T},{\hat{z}}_{S},u\right)=-\sum_{i}u_{i}\sigma {\left({\bar{z}}_{T},T\right)}_{i}\left\{\log\left(\sigma {\left({\hat{z}}_{S},T\right)}_{i}\right)-\log\left(\sigma {\left({\bar{z}}_{T},T\right)}_{i}\right)\right\},$$

where σ(z,T) is the softmax function with the temperature *T*, and $\sigma {\left(z,T\right)}_{i}$ is the *i*-th component of σ(z,T). In (9), $\sigma {\left(z,T\right)}_{i}$ can be computed as

(10) $$\sigma {\left(z,T\right)}_{i}=\frac{\exp(z_{i}/T)}{\sum_{j}\exp\left(z_{j}/T\right)}.$$

**Figure 4:**
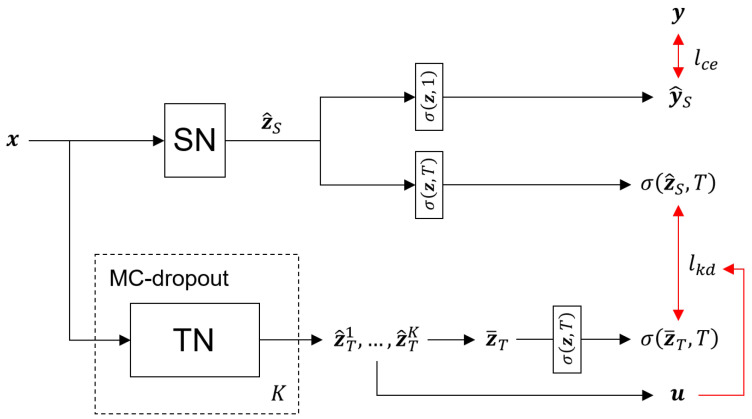
The procedure of uncertainty-aware knowledge distillation for the training of the student network; SN and TN indicate the student and teacher networks, respectively, and σ(z,T) is the softmax function with the temperature *T*.

The overall procedure for the training of the student network is presented in Figure 4.

### 4.3. Post-Processing

**Figure 5:**
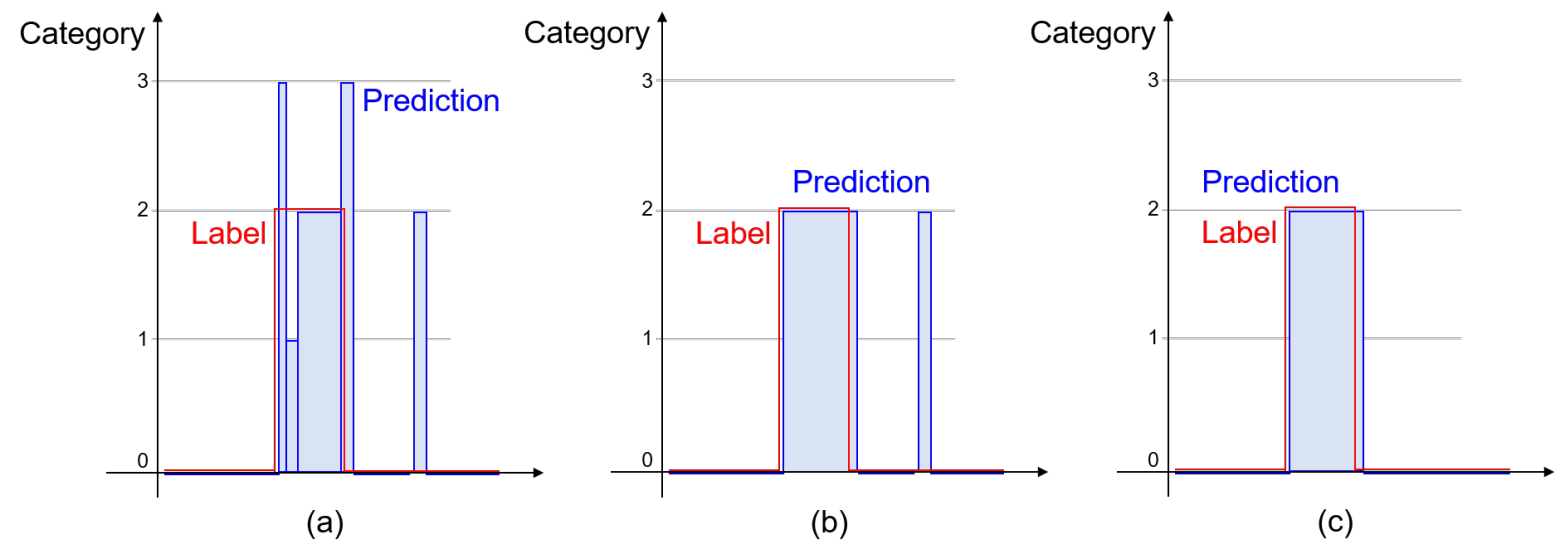
The procedure for the post-processing. (**a**) presents the predictions from the student network, and (**b**) presents the result of grouping non-zero connected samples and assigning an identical category of the maximum frequent. (**c**) presents the result of a thresholding method.

The post-processing to reduce errors in network predictions is inspired by a connected component analysis in image-processing techniques. In the labeled data, a collision is represented by connected samples, with a non-zero number corresponding to its location. However, a few predictions may differ from their adjacent predictions, because a neural network independently infers predictions for different data samples. Based on the collision properties in the labeled data, incorrect predictions are reduced by the post-processing presented in Figure 5.

**Figure 6:**
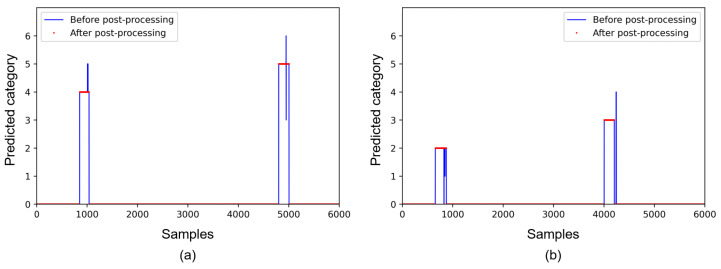
Examples of predictions before and after the post-processing. (**a**) presents predictions for the collision categories of 4 and 5, and (**b**) presents predictions for the collision categories of 2 and 3.

The post-processing is composed of two steps; in Figure 5, (a) shows predictions from the student network, and (b) and (c) present the results after the first and second post-processing steps, respectively. In the first step, non-zero connected samples are grouped, and the number of samples for each category are counted. Predictions in a group are replaced into the category which corresponds to the maximum frequency, as presented in Figure 5b. In the second step, if the number of non-zero connected samples is less than a threshold value, then these samples are regarded as the normal state. The threshold value of 10 samples is utilized in experiments, and Figure 6 presents examples of the results of the post-processing.

## 5. Experiments

### 5.1. Experimental Environment and Evaluation Measures

The proposed algorithm is developed within a hardware environment including Intel core i7-10700 CPU, 32GB DDR4 RAM, and RTX 3080 GPU. In experiments, Python and Pytorch are mainly utilized to implement the proposed algorithm and to conduct an ablation study. To demonstrate the proposed method, the dataset is gathered from a collaborative robot, which consists of six rotating joints. The cobot weighs 47 kg, has a maximum payload of 10 kg, and reaches up to 1300 mm. The actuator consists of motors manufactured from Parker, motor drivers from Welcon, and embedded joint torque sensors in each joint. The hardware of the cobot contains a custom embedded controller, based on real-time linux kernel, and it communicates with drivers through EtherCAT with a cycle time of 1 ms.

To demonstrate the effectiveness of the proposed method, we evaluate the algorithm in three ways: (1) sample-level accuracy, (2) collision-level accuracy, and (3) time delay. In the process of collision identification, deep neural networks perform sample-level multiclass classification, which classifies each sample, composed of a 24-dimensional sequence of sensor data, into the normal state or one of six collision locations. To evaluate the sample-level accuracy of deep neural networks, we measure Recall, Precision, and F1-score for each sample, which are defined as follows:

(11) $$\begin{array}{cc} Recall & =TP/(TP+FN),\\ Precision & =TP/(TP+FP),\\ F1-score & =2\times \frac{precision\times recall}{precision+recall}, \end{array}$$

where TP, FP, FN are the numbers of true positives, false positives, and false negatives, respectively. True positive is a correctly identified collision sample, false positive is an incorrect prediction, which is classified into a collision, and false negative is an incorrect prediction which is classified into the normal state.

**Figure 7:**
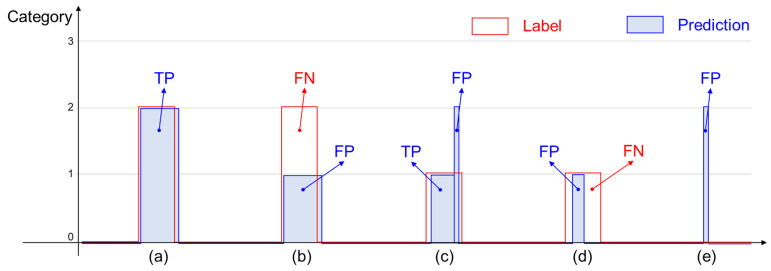
Examples of true-positive, false-positive, and false-negative collisions for computing collision-level accuracies. (**a**) presents a TP collision, (**b**,**d**) present FP and FN cases, (**c**) presents TP and FP cases, and (**e**) presents a FP collision.

Collision-level accuracy is another important measure for evaluating a collision identification system. Because collaborative robots respond to each collision, reducing the number of false positive collisions is an important issue. Recall, Precision, and F1-score are computed as (11) with different definitions of TP, FP, and FN to measure the collision-level accuracy. A group of connected samples that are classified into a collision is regarded as a true positive if the intersection over union (IoU) between the connected predictions and its corresponding true collision samples is greater than 0.5. A group of predictions that are classified into a false category of collisions is regarded as a false positive, and a false negative is a missed collision. Figure 7 shows several cases of TP, FP, and FN for measuring the collision-level accuracy.

Finally, the time delay is measured to evaluate the processing time of the collision identification system. For the safe and reliable collaborations of human and robots, the processing time is required to be reduced as possible. The total processing time is composed of the inference time of a neural network, detection delay for collisions, and post-processing time. Based on these three types of evaluation measure, the effectiveness of the proposed method is demonstrated in experiments.

### 5.2. Training of Neural Networks

**Figure 8:**
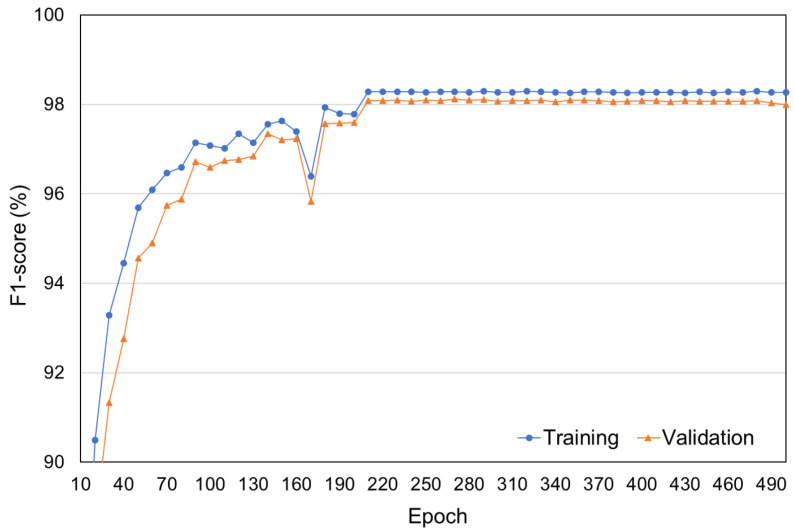
F1-scores for the training and validation datasets.

To train the neural networks, Adam optimizer [63] is utilized with a learning rate of $10^{-4}$. The learning rate is decreased to $10^{-5}$ after training 200 epochs. Figure 8 presents f1-scores for the training and validation datasets during the training of 500 epochs. As shown in Figure 8, after training a sufficientl number of epochs, the validation accuracy was not further decreased. Therefore, in the following experiments, the accuracies of deep neural networks are evaluated for the test set after training 300 epochs.

**Figure 9:**
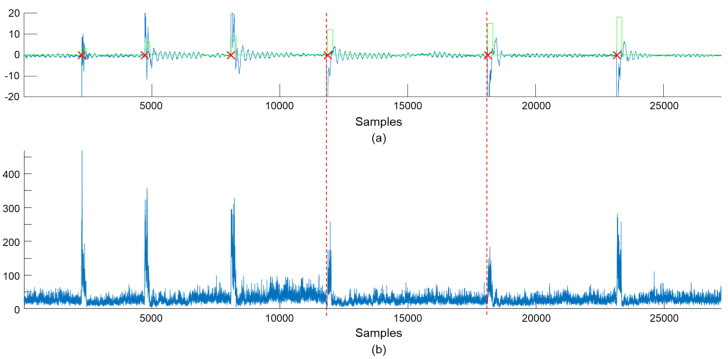
Uncertainties measured by MC-dropout of the teacher network. (**a**) shows the first dimension of 24-dimensional sensor data, and (**b**) presents uncertainties measured by MC-dropout. In (**a**), red × marks indicate collision moments, and green lines represent labels for the normal state and locations of collisions.

To train the student network, the temperature of the softmax function is set to 5 during the process of knowledge distillation. The temperature value has to be greater than 1 to soften probabilistic predictions of neural network, and temperature values between 2 and 5 are usually used for knowledge distillation in the previous literature [39]. In our experiments, modifications to the temperature value glead to insignificant changes in the experimental results. In Figure 9, (a) shows the first dimension of 24-dimensional sensor data, which corresponds to the torque signal at the first joint, and (b) presents uncertainties measured by MC-dropout with the value of K=4. As shown in Figure 9, the uncertainties of collision samples are high compared to normal state samples. By weighting the uncertainties on the KL-divergence between probabilistic predictions of the student and teacher network, the student network is able to focus on learning difficult data samples.

### 5.3. Sample-Level Accuracy

The first measure to evaluate the performance of deep neural networks is the sample-level accuracy. As explained in Section 4.1, the architecture of the deep neural network proposed in [22] is employed to construct the base model. To demonstrate the effectiveness of uncertainty-aware knowledge distillation for the problem of collision identification, we compare the accuracies of the proposed method with those of the base model and a student network. The student network has an identical architecture to the base model, and is trained by distilling knowledge in the teacher network without employing uncertainty information. Table 2 presents the sample-level recall, precision, and f1-score of four neural network models; the proposed method means another student network, which is trained by uncertainty-aware knowledge distillation. The last row of Table 2 presents the sample-level accuracies of the teacher network. As presented in Table 2, the f1-scores of the proposed method are comparable to those of the teacher network; it is worth noting that the proposed method employs a lightweight network compared to the teacher network.

### 5.4. Collision-Level Accuracy

This section presents the collision-level accuracies. As collaborative robots react to each collision, reducing the number of false-positive collisions is a very important issue in reliable collision identification systems. In the labeled data, a collision is represented by 200 non-zero samples; therefore, false-positive collisions, which are composed of a few fals- positive samples, are not effectively reflected in the sample-level accuracies. Although the sample-level accuracies of the four neural network models are above 98%, there are a considerable number of false-positive collisions. To compute the collision-level accuracies, a group of non-zero predictions is regarded as a collision, and Table 3 presents the numbers of true-positive, false-positive, and false-negative collisions of the four neural network models. In Table 3, the base model, student network, and proposed method have an identical network architecture to CollisionNet [22]; the student network is trained by regular knowledge distillation, and the proposed method employs uncertainties during knowledge distillation. As shown in Table 3, the number of false positives is significantly reduced after the post-processing. Table 4 presents the collision-level recall, precision, and f1-score of the four neural networks. By utilizing probabilistic labels and uncertainties from the teacher network, the proposed method produces better accuracies, despite its lightweight network architecture compared to the teacher network.

### 5.5. Analysis for the Processing Time

The processing time is another important factor for responding to external forces within an acceptable timeframe. In the collision identification system, the total processing time is composed of the inference time of a neural network, time delay for detecting a collision, and post-processing time. Table 5 presents the averaged processing time for each step. The teacher network requires an 83% longer inference time compared to the base model, student network, and proposed method. The detection delay is measured by averaging the time intervals between collision occurrences and their corresponding first true-positive samples. As presented in Table 5, the proposed method requires 2.6350 ms to identify a collision occurrence, and this satisfies the requirement for the safety of collaborative robots.

## 6. Conclusions

This paper proposes a collision identification method for collaborative robots. To identify the locations of external forces, the propose method employs a deep neural network, which is composed of causal convolutions and dilated convolutions. The key contribution is the method of capturing sample-level uncertainties and distilling the knowledge of a teacher network to train a student network, with consideration of predictive uncertainties. In the knowledge distillation, KL-divergence between the predictions of the student and teacher networks are weighted by the predictive uncertainties to focus on data samples that are difficult to train. Furthermore, we also propose a post-processing to reduce the number of false-positive collisions. Experiments were conducted with a 6-DoF-articulated robot, and we demonstrated that the uncertainty is beneficial to improving the accuracy of the collision identification method.

## Figures and Tables

**Table 1:** The number of collisions and data samples. *Total* indicates the number of data samples, which were collected with a sampling rate of 1 kHz, and *Collision* indicates the number of collision samples.

	Training Set		Validation Set		Test Set	
Collisions	3906		558		1122	
Samples	*Total*	*Collision*	*Total*	*Collision*	*Total*	*Collision*
	19,563,048	781,200	2,778,777	111,600	5,798,685	224,400

**Table 2:** Sample-level accuracies of the four different neural network models before and after the post-processing.

	Before Post-Processing			After Post-Processing		
	Recall	Precision	F1-Score	Recall	Precision	F1-Score
Base model	98.1611	98.3985	98.2796	98.5473	99.0617	98.8038
Student network	98.2015	98.3458	98.2736	98.5992	99.0198	98.8091
Proposed method	98.3110	98.4516	98.3812	98.7119	99.0465	98.8789
Teacher network	98.2729	98.5337	98.4031	98.5629	99.1011	98.8313

**Table 3:** The numbers of true-positive (TP), false-positive (FP), and false-negative (FN) collisions of the four neural network models before and after post-processing.

	Before Post-Processing			After Post-Processing		
	TP	FP	FN	TP	FP	FN
Base model	1119	229	3	1119	121	3
Student network	1118	295	4	1118	109	4
Proposed method	1120	205	2	1120	76	2
Teacher network	1119	267	3	1119	77	3

**Table 4:** Collision-level accuracies of the four different neural network models before and after the post-processing.

	Before Post-Processing			After Post-Processing		
	Recall	Precision	F1-Score	Recall	Precision	F1-Score
Base model	99.7326	78.9139	88.1102	99.7326	90.2419	94.7502
Student network	99.6436	79.1224	88.2052	99.6435	91.1165	95.1894
Proposed method	99.8217	84.5283	91.5406	99.8217	93.6454	96.6350
Teacher network	99.7326	80.7359	89.2344	99.7326	93.5619	96.5487

**Table 5:** The averaged processing time in ms for the collision identification.

	Inference Time	Detection Delay	Post-Processing	Total
Base model	1.7641	0.8239	0.2057	2.7938
Student network	1.7641	0.6198	0.2057	2.5897
Proposed method	1.7641	0.6651	0.2057	2.6350
Teacher network	3.2348	0.7006	0.2057	4.1412
